# Vagus nerve stimulation for treating developmental and epileptic encephalopathy in young children

**DOI:** 10.3389/fneur.2023.1191831

**Published:** 2023-10-20

**Authors:** Guifu Geng, Wandong Hu, Yao Meng, Huan Zhang, Hongwei Zhang, Chuanmei Chen, Yanqing Zhang, Zaifen Gao, Yong Liu, Jianguo Shi

**Affiliations:** ^1^Department of Epilepsy Center, Children's Hospital Affiliated to Shandong University, Jinan Children's Hospital, Jinan, Shandong, China; ^2^Department of Functional Neurosurgery, Children's Hospital Affiliated to Shandong University, Jinan Children's Hospital, Jinan, Shandong, China; ^3^Pediatric Health Care Institute, Children's Hospital Affiliated to Shandong University, Jinan Children's Hospital, Jinan, Shandong, China

**Keywords:** developmental and epileptic encephalopathy, vagus nerve stimulation, gene mutation, cognitive disorder, drug-resistant epilepsy

## Abstract

**Objective:**

To investigate the clinical variables that might predict the outcome of developmental and epileptic encephalopathy (DEE) after vagus nerve stimulation (VNS) therapy and identify the risk factors for poor long-term outcome.

**Patients and methods:**

We retrospectively studied 32 consecutive children with drug-resistant DEE who had undergone VNS surgery from April 2019 to July 2021, which were not suitable for corpus callosotomy. In spite of combining valproic acid, levetiracetam, lamotrigine, topiramate, etc. (standard anti-seizure medicine available in China) it has not been possible to effectively reduce seizures in the population we investigate (Cannabidiol and brivaracetam were not available in China). A responder was defined as a frequency reduction decrease > 50%. Seizure freedom was defined as freedom from seizures for at least 6 months. Sex, electroencephalograph (EEG) group, neurodevelopment, time lag, gene mutation, magnetic resonance imaging (MRI), and epilepsy syndrome were analyzed with Fisher's exact test, The age at onset and age at VNS therapy were analyzed with Kruskal-Wallis test, statistical significance was defined as *p* < 0.05. And used the effect size to correction.

**Results:**

Among the 32 patients, the median age at VNS implantation was 4.7 years (range: 1–12 years). At the most recent follow-up, five children (15.6%) were seizure-free and 22 (68.8%) were responders. Univariate analysis demonstrated that the responders were significantly associated with mild development delay/intellectual disability (*p* = 0.044; phi coefficient = 0.357) and a multifocal EEG pattern (*p* = 0.022; phi coefficient = −0.405). Kaplan-Meier survival analyses demonstrated that a multifocal EEG pattern (*p* = 0.049) and DEE without epileptic spasm (ES) (*p* = 0.012) were statistically significant (*p* = 0.030). Multivariate analysis demonstrated that DEE with ES had significant predictive value for poor long-term outcome (*p* = 0.014, hazard ratio = 5.433, confidence interval = 1.402–21.058).

**Conclusions:**

Our study suggested that VNS was a generally effective adjunct treatment for DEE. Although the predictive factors for VNS efficacy remain unclear, it should be emphasized that patients with ES are not suitable candidates for epilepsy surgery. Further investigations are needed to validate the present results.

## Background

The concept of developmental and epileptic encephalopathy (DEE) was first proposed by the International League Against Epilepsy (ILAE) in 2017 (1). Many DEEs are related to gene variants and begin in the early infantile period, such as early infantile DEE (EIDEE), epilepsy in infancy with migrating focal seizures (EIMFS), and infantile epileptic spasms syndrome (IESS) (2, 3). The development of molecular genetics has linked an increasing number of genes to DEE. One study demonstrated that among children in China with epilepsy onset within the first year of life, 28.2% of neonates and 34.8% of infants had a genetic diagnosis (4). Examples of a genetic link include epilepsy associated with *KCNQ2, CDKL5*, and *SCN1A* mutations (5, 6).

Individuals with DEE have high rates of comorbid conditions, which include intellectual disability (ID), autism spectrum disorder (ASD), and behavioral issues (7, 8). In such cases, outcomes remain poor even when the seizures stop (1). In genetically associated DEE, seizures might occur earlier than cognitive impairment (9) and might aggravate cognitive disorder, where seizure treatment might likely improve neurodevelopment. Many DEEs begin in early infancy, which is a crucial neurodevelopment period. Therefore, effective therapeutic intervention should be considered early in DEEs (8, 10).

In most cases, DEEs are highly resistant to treatment and associated with unfavorable outcomes, both in relation to seizures and to cognitive disorder (11). Vagus nerve stimulation (VNS) has been extensively used for treating DEE (12, 13). In 2000, the China Food and Drug Administration (CFDA) approved VNS as an adjunct treatment for intractable epileptic seizures in adults and children. VNS is a safe and effective neuromodulatory therapy for pediatric medically refractory epilepsy, where the response rates (>50% seizure reduction) are ~35–57% after 2-year follow-up and are higher at ≥5-year follow-up (14, 15). A higher seizure reduction rate was reported in children aged < 6 years (16). Furthermore, VNS treatment was associated with improvement in motor, language, and personal/social development in children (17). A previous study demonstrated that VNS therapy improved cognitive disorder, autism and quality of life (QOL) in low-IQ patients (18). Furthermore, VNS was effective in pediatric patients with drug-resistant epilepsy (DRE) of monogenic etiology (19, 20). In *CDKL5*-DEE, seizure improvement was reported in more than two-thirds of patients post-VNS therapy, and additional benefits such as cognitive and behavioral improvements justify further use in other gene-induced DEEs (21).

Currently, the benefits of VNS in DEE are not well defined. In this article, we present a retrospective analysis of a series of patients with DEE who underwent VNS therapy. The main study goals were to: (1) investigate clinical variables that might predict the DEE outcome post-VNS therapy and (2) identify risk factors for poor long-term outcome.

## Methods

### Preoperative patient evaluation

We retrospectively studied 32 consecutive children (17 girls, 15 boys) with DEE who had undergone VNS surgery (G112, PINS Medical, Beijing, China) and had been admitted to Children's Hospital Affiliated to Shandong University from April 2019 to July 2021. The inclusion criteria were: (1) age < 12 years at VNS therapy (Many kinds of antiepileptic drugs were taken before surgery, but the effect was not good; Mean while not suitable for corpus callosotomy); (2) diagnosis of drug-resistant DEE according to ILAE-defined criteria (include Early-infantile developmental and epileptic encephalopathy, Dravet syndrome, IESS, Etiology-specific syndromes, Lennox-Gastaut Syndrome and others) (22); (3) follow-up period of at least 1 year post-VNS therapy. Patients were excluded if they had undergone epilepsy surgery or the ketogenic diet (KD) before or during VNS therapy.

All children underwent brain magnetic resonance imaging (MRI). Standard MRI was performed on a 3.0 Tesla SP system (Siemens, Erlangen, Germany) using standardized epilepsy protocols that included high-resolution T1-weighted volume acquisition, T2-weighted, and fluid attenuated inversion recovery (FLAIR) sequences. The MRI findings were classified into normal or abnormal groups.

All children underwent 3-h electroencephalograph (EEG) recording pre-VNS therapy. The EEGs were recorded while the patients were asleep and awake. The EEG findings were classified into focal or multifocal epileptiform discharges or generalized epileptiform discharges groups. All children were evaluated at a multidisciplinary team (MDT) conference to determine their treatment strategies. This study was approved by the ethical committee of Children's Hospital Affiliated to Shandong University. Each patient's parents signed the informed consent form. The epilepsy syndromes were encoded according to the ILAE (23). The epilepsy syndrome groups were classified into IESS, LGS and Others. The patients' anti-seizure medicines (ASMs) were not changed during the first 3 months post-surgery.

The time lag referred to the delay between clinical seizure onset and VNS treatment initiation. The patients were divided into short (<3 years) or long (≥3 years) time lag groups.

### Neuropsychological assessment

Neurodevelopment and neuropsychological data were collected retrospectively. Neurodevelopment was assessed before treatment with the Chinese Child Developmental Behavior Assessment Scale. The patients were assigned to three groups: mild development delay/ID (DD/ID) group (independent movement and communication), moderate DD/ID (independent movement and partial communication), and severe DD/ID (completely dependent movement and no communication). Their QOL was assessed by questionnaires and visual analog scales completed by their parents.

### Outcome evaluation

The baseline seizure frequency (seizures/month) was evaluated pre-VNS device implantation. A 3-month baseline seizure frequency was used for comparison with post-VNS. A responder was defined as a frequency reduction decrease > 50%. Seizure freedom was defined as freedom from seizures for at least 6 months.

### VNS programming

The VNS generator was implanted in the left side of the chest using previously described techniques. In each child, the VNS stimulator was switched on 7 days post-implantation. The stimulation parameters in the conventional mode at startup were as follows: amplitude, 0.2 mA; pulse width, 250 μs; frequency, 30 Hz; stimulation duration, 30 s; and interval, 5 min. The pulse amplitude was increased by 0.2–0.3 mA every 2 weeks and gradually adjusted to 1.5–2.0 mA. Duty cycles (ON and OFF times) were adjusted after 6–8 months if no improvement was observed.

### Statistical analysis

Statistical analysis was performed using SPSS Statistics 23 (IBM Corp., Armonk, NY, USA). Normally distributed continuous variables are described using the median and the mean and standard deviation (SD). Sex, EEG group, neurodevelopment, time lag, gene mutation, MRI, and epilepsy syndrome were analyzed using Fisher's exact test. The age at onset and age at VNS therapy were analyzed with Kruskal-Wallis test. Statistical significance was defined as *p* < 0.05. And used the effect size to correction. The probability of responders was calculated with Kaplan–Meier survival analysis. Statistical significance was tested using the log-rank test and comparison of 95% confidence intervals (CIs). An additional multivariate Cox proportional hazard regression model was used to determine independent variables that could be used as predictors of poor responders. Results were considered statistically significant at the 5% level.

## Results

### Patients' characteristics

A total of 43 children underwent VNS implantation and 32 children fulfilled the study inclusion criteria. Among the 32 children, the median age at VNS implantation was 4.7 years (range: 1–12 years) and 53% were female (*n* = 17). The median age at seizure onset was 22.37 months (range: 1 day−96 months) and a median of five ASMs had been attempted previously (range: 2–8). The five most commonly used ASMs were valproic acid, levetiracetam, lamotrigine, topiramate and clonazepam (Table 1, Because we didn't have new ASMs as cannabidiol, brivaracetam in China). The mean stimulation parameters at 6 months was 1.2 mA (range: 0.4–1.7) and 30 s on−5 min off (10% duty cycle). Meanwhile the mean stimulation parameters at last was 1.3 mA (range: 0.2–2.0) and 30 s on−5 min off (10% duty cycle).

**Table 1:** DEE patient anti-seizure medicines.

**Characteristics**	**Anti-seizure medicines pre-VNS**	**Anti-seizure medicines post-VNS**
Patient 1	VPA, LEV, OXC, CZP, LTG, KD	LCM, CZP, LTG
Patient 2	LEV, VPA, CZP, TPM, LTG	LEV, VPA
Patient 3	LEV, OXC, VPA, CZP, TPM, LTG, LCM	OXC
Patient 4	LEV, OXC, LCM	LEV, OXC
Patient 5	ACTH, OXC, VPA, TPM, VGB, RAPA	OXC, VPA
Patient 6	LEV, OXC, VPA, CZP, TPM, LTG, LCM	VPA, CLB, LTG
Patient 7	LEV, VPA, CZP, TPM, LTG, CLB	None
Patient 8	LEV, OXC, VPA, CZP, TPM, KD	LEV, VPA, PER
Patient 9	LEV, VPA, CZP, TPM, LTG	PER, VPA, CLB, LTG
Patient 10	LEV, OXC, VPA, TPM	VPA, PER
Patient 11	LEV, VPA, CZP, TPM	LEV, VPA, PER
Patient 12	VPA, TPM, VGB, PER	VPA, TPM, VGB, CLB
Patient 13	LEV, VPA, CZP, TPM	LEV, VPA
Patient 14	LEV, VPA, CZP, TPM, LTG, PER	ZNS, PB
Patient 15	ACTH, LEV, VGB, VPA, CLB, TPM	CLB, OXC, LTG
Patient 16	PB, LEV, OXC, VPA, TPM	LEV, OXC, TPM
Patient 17	ACTH, VPA, CZP	VPA, PER
Patient 18	ACTH, ZNS, VPA, Nitrazepam, TPM, LTG, PER, CLB	VPA, Nitrazepam, LTG
Patient 19	LEV, VPA, TPM	LEV, VPA, TPM, CZP
Patient 20	VPA, TPM, LTG, PER	VPA, TPM, LTG, PER
Patient 21	VPA, TPM	VPA, TPM
Patient 22	LEV, VPA, PER	PER, TPM
Patient 23	ACTH, LEV, OXC, VPA, CZP, TPM, LCM, KD	OXC, PER, CLB
Patient 24	ACTH, LEV, VGB, VPA, CLB, TPM, LTG, ZNS, PER	VPA, LTG
Patient 25	VPA, CLB, LTG, ZNS	VPA, CLB, LTG
Patient 26	ACTH, KD, LEV, ZNS, VPA, CZP, TPM, LTG, PER	LEV, ZNS, VPA, CZP, TPM
Patient 27	LEV, OXC, VPA, TPM	LEV, OXC, VPA, TPM
Patient 28	LEV, VPA, TPM, LTG, PER	LEV, VPA, TPM, LTG
Patient 29	OXC, VPA, CZP, TPM, LTG	TPM, LTG, LCM
Patient 30	LEV, VPA, CZP, TPM, KD	VGB
Patient 31	LEV, VPA, CZP, TPM, PER	CLB
Patient 32	Methylprednisolone, LEV, VPA, CLB, CZP	LEV, LTG

LEV, Levetiracetam; VPA, Valproate; OXC, Oxcarbazepine; TPZ, Topiramate; PER, Perampanel; CLB, clobazam; KD, ketogenic diet; CZP, Clonazepam; LTG, Lamotrigine; VGB, Vigabatrin; ZNS, Zonisamide; LCM, Lacosamide; PB, Phenytoin sodium.

Twenty-two of the 32 children underwent genetic testing. Of these 22 children, 10 were diagnosed with monogenic epilepsy in which the pathogenic genes were *SCN1A* (*n* = 4), *KCNQ2* (*n* = 2), *KCNT1* (*n* = 1), *STXBP1* (*n* = 1), *TSC1* (*n* = 1), and *COL4A1* (*n* = 1). According to the new DEE classification, 12 children were diagnosed with IESS, six with Lennox-Gastaut syndrome (LGS), four with Dravet syndrome (DS), one with DEE with spike-and-wave activation in sleep, one with EIMFS, one with EIDEE, and seven with unclassified DEE. All children underwent video-EEG, where 16 children (50%) each demonstrated multifocal epileptiform discharges, others showed diffuse or generalized epileptiform discharges in interictal EEG.

Sixteen children (50%) had abnormal 3-Tesla MRI scans: nine had atrophy associated with delayed myelination, two had pachygyria, two had cortical malformations, one had tuberous sclerosis, one had bilateral subcortical band heterotopia, and one had thin corpus callosum with ventriculomegaly. Eight children underwent interictal positron emission tomography (PET): two were normal, five had diffuse or multifocal hypometabolism, and one had hemispheric hypometabolism.

Development assessment demonstrated that only four children (12.5%) were normal before seizure onset. All children demonstrated DD pre-VNS therapy: 19% (6/32) had mild DD/ID, 53% (17/32) had moderate DD/ID, and 28% (9/32) had severe DD/ID.

Comparison of the changes in the QOL at pre-VNS and after at least 12 months of VNS therapy revealed that 5/32 children (15.6%) reached new milestones or acquired new skills post-VNS treatment. Behavior, mood, verbal communication improvement and progress with schoolwork were reported by 56.25% of the parents (18/32) during follow-up.

### Response to VNS treatment

All children had at least 12 months of follow-up [mean follow-up: 22 (range: 12–47) months]. At 6 months post-VNS implantation, 13 children (40.6%) were responders and one child (3.1%) was seizure-free. At the most recent follow-up, five children (15.6%) were seizure-free and 22 (68.8%) were responders. The age at DEE onset was ≤12 months in 16 children (50%) while the time lag was <3 years in 20 children (62.5%).

The Chi-square test and univariate analysis with a two-tailed Fisher's exact test determined that the age at seizure onset, time lag, age at implantation, sex, epilepsy syndrome, and MRI scan were not significantly different between the responder and non-responder groups. Responders were significantly associated with mild DD/ID (*p* = 0.044) and a multifocal EEG pattern (*p* = 0.022; Table 2). The time lag was <3 years in most children in the responder group (16/22) as compared to the non-responder group (4/10), but no statistical difference was detected. The phi coefficients of sex, developmental delay and seizure type were all in the range of 0.3–0.6, so the degree of association between them and prognosis was moderately positive. The phi coefficients of time interval and EEG ranged from −0.3 to −0.6, and they were moderately negatively correlated with prognostic factors, while the remaining variables were weakly correlated with prognostic factors, especially genes and ASMs. Table 2 presents the patients' preoperative data.

**Table 2:** DEE patient characteristics (*n* = 32).

**Characteristics**	**All patients**	**Responders**	**Non-responders**	***p***	**phi coefficient**	**Coefficient of dependence**
	***N*** = **32**	***N*** = **22**	***N*** = **10**			
Sex, female: male	17:15	14:08	3:07	0.077	0.312	0.298
Age at onset, Mean ± SD, months	22.37 ± 24.70	22.05 ± 24.09	22.05 ± 27.33	0.76		
Age at VNS, Mean ± SD, years	4.78 ± 2.76	4.63 ± 2.84	5.10 ± 2.68	0.497		
Number of ASMs before VNS				0.844	0.035	0.035
≤ 4	12	8	4			
>4	20	14	6			
Time lag				0.076	−0.313	0.299
≤ 3 years	20	16	4			
>3 years	12	6	6			
Gene mutation				0.918	−0.018	0.018
Yes	10	7	3			
Not clear	22	15	7			
Development delay before VNS				0.044	0.357	0.336
Mild	7	15	10			
Others	25	7	0			
MRI				0.127	0.270	0.260
Normal	16	9	7			
Abnormal	16	13	3			
EEG				0.022	−0.405	0.375
Multifocal	16	14	2			
Diffuse or general	16	8	8			
Epilepsy syndrome				0.177	0.329	0.313
IESS	12	7	5			
LGS	6	3	3			
Others	14	12	2			

DEE, developmental and epileptic encephalopathy; MRI, Magnetic Resonance Imaging; EEG, electroencephalogram; IESS, infantile epileptic spasms syndrome; LGS, Lennox-Gastaut syndrome.

**Figure 1:**
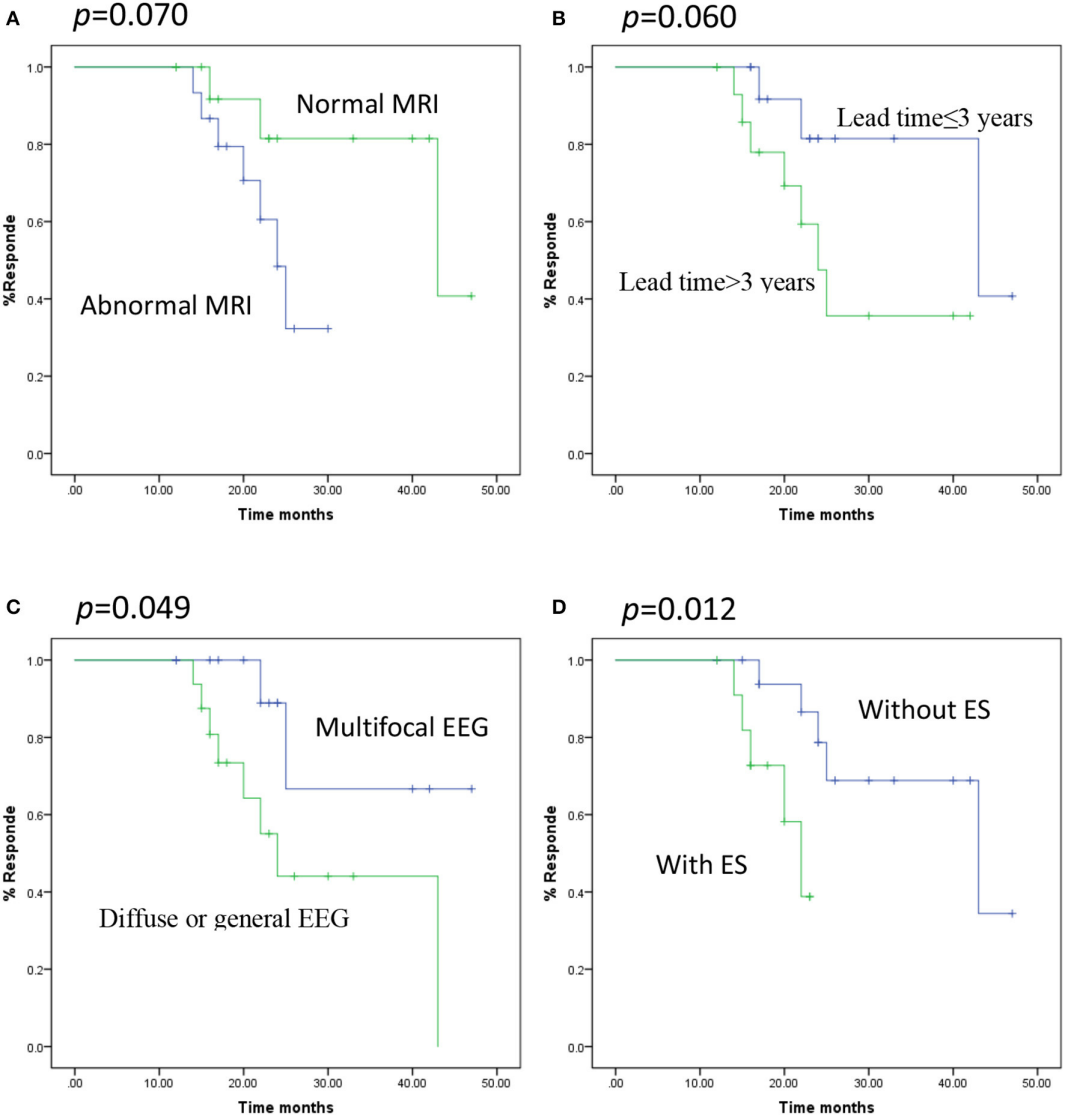
Kaplan-Meier curves showing distinct respond depending on the various predictors. **(A)** MRI, normal vs. abnormal (log-rank test *p* = 0.070), **(B)** time lag, ≤3 vs. >3 years (log-rank test *p* = 0.060), **(C)** EEG pattern, Multifocal vs. Diffuse or general (log-rank test *p* = 0.049), **(D)** seizure type, ES vs. without ES (log-rank test *p* = 0.012).

Survival analyses were conducted with the Kaplan-Meier method and proportional hazards regression. A multifocal EEG pattern (*p* = 0.049) and DEE without epileptic spasm (ES) (*p* = 0.012) had significant predictive value for favorable seizure outcome at 22 months (seizure reduction > 50%) (*p* = 0.030; Figure 1). The multivariate analysis (multivariate Cox proportional hazard regression model) demonstrated that neurodevelopment, age at onset, time lag, MRI, and EEG pattern lost their predictive value and that only DEE with ES had significant predictive value for poor long-term outcome (*p* = 0.014, hazard ratio = 5.433, CI = 1.402–21.058) (Table 3).

**Table 3:** Multivariate analysis predictors of recurrence by multivariate Cox proportional hazard regression model.

**Predictor**	***p-*value**	**HR**	**95% CI**
**Neuro development delay**			
Mild vs. others	0.599	0.492	0.035–6.914
**Age at onset**			
≤ 12 vs. >12 months	0.381	0.367	0.039–3.455
**Time lag**			
≤ 3 vs. >3 years	0.117	6.619	0.622–70.451
**MRI**			
Normal vs. others	0.180	0.199	0.019–2.109
**EEG**			
Multifocal vs. Others	0.288	2.500	0.462–13.536
**Seizure type**			
ES vs. without ES	0.014	5.433	1.402–21.058

vs., versus; HR, hazard ratio; CI, confidence interval; MRI, Magnetic Resonance Imaging; EEG, electroencephalogram; ES, epileptic spasms.

### Adverse effects

No significant complications were reported. Two children complained of coughing and hoarseness when the stimulation parameters were increased. In one case, the VNS was switched off due to poor efficacy.

## Discussion

In 2022, the ILAE classified DEE as an epilepsy syndrome with onset in neonates and infants (23). The term “DEE” refers to cognitive functions being influenced by both seizure and interictal epileptic discharge and the neurobiological process behind the epilepsy. Approximately 50% of DEEs have genetic etiology (24, 25). Abnormal neurological behavior or development often presents before seizure onset (9), where we demonstrated that only four children (12.4%) exhibited normal behavior before seizure onset. The interictal EEG pattern and maturational state of the brain influence the effect of epileptic activity on cognition and development (11).

The long-term seizure control rate for DEEs is poor (26). VNS is a well-tolerated and effective adjunctive treatment for medically intractable DEE (20, 27). Although the mechanisms of VNS are not well understood, it is thought that VNS activates the release of epinephrine and norepinephrine (NE) in the locus coeruleus (LC), which may subserve cognitive enhancement and memory improvement (28). The antiseizure effects of VNS may also relate to altered activity in the limbic system, autonomic networks and reticular activating system (15). In our study, 68.8% of the children (22/32) experienced at least 50% reduction in seizure frequency and 15.6% (5/32) had achieved seizure-free status at the last follow-up. The seizure reduction rate was similar to that reported previously (14, 20).

Age at implantation, duration of epilepsy, etiology, and seizure type might be predictive factors of VNS efficacy (29, 30). However, the predictive factors of VNS efficacy in DEE remain unclear. As clearly demonstrated in our study, a multifocal EEG pattern was a predictor for responders. In patients with post-traumatic epilepsy and post-encephalitic epilepsy, focal or multifocal EEG patterns also indicated better efficacy post-VNS therapy (31, 32). However, another study reported that a generalized epilepsy group demonstrated the best response in seizure reduction (33). Our data demonstrated a significant difference in the number of responders between children with better (mild) and worse (moderate and severe) DD/ID, where 100% of responders were from the mild DD/ID group. Although recent studies confirmed that seizure reduction in DD/ID patients was similar to that in a non-DD population (34), subgroup analysis was not performed for these patients.

VNS implantation at age ≤ 2 years was strongly associated with better developmental and cognitive outcome, and QOL (35). Younger age at implantation suggests a shorter duration of epilepsy, where several studies demonstrated that a short duration of epilepsy before VNS implantation was a strong factor associated with good outcome (29, 36). By contrast, other studies reported that a shorter epilepsy duration did not predict a better outcome (37, 38), which was similar to our findings.

We determined that DEE with ES had significant predictive value for poor long-term response (*p* = 0.014, hazard ratio = 5.433, CI = 1.402–21.058). The result was similar to that of an earlier study, where only two of the 16 (13%) patients in the ES group demonstrated seizure reduction > 50% (39). The efficacy of VNS for treating ES was poor when compared to that for treating other syndromes. For example, a meta-analysis of 480 patients with LGS suggested that 54% of the patients responded to VNS therapy (12). Previous clinical study showed that in patients with ES, the thalamus and brainstem might already be sufficiently activated to suppress the seizures. So, additional stimulation of the brainstem following VNS might be unable to generate further inhibitory effects (40).

Most common delayed post-operative effects in people treated with VNS are hoarseness, voice alteration (usually happening during VNS activation), dyspnoea and post-operative pain. Stimulation-dependent adverse effects VNS is commonly associated with adverse effects attributable to the local effect of current (15).

During the follow-up, 56.25% of the parents (18/32) reported improved QOL, which paralleled recent previous studies (41). The precise mechanism of VNS treatment remains unknown. There is much evidence for the effects of VNS on pediatric neurodevelopmental and other psychiatric disorders (42, 43). The ability of VNS to improve development with a benign adverse effect profile encouraged us to pursue it as a treatment option in children with DEE. VNS should be considered an option for treating refractory epilepsy in this population before additional ASMs are prescribed.

## Limitations

First, the number of DEE cases was small. Second, part of the clinical data were retrospective, and accurate determination of seizure frequency was difficult. Therefore, the influence of VNS on the neuropsychological aspect requires further investigation.

## Conclusions

Our findings suggested that VNS was a generally effective adjunct treatment for DEE. The onset of many DEEs occurs in neonatal and/or early infancy, which is a crucial neurodevelopment period. Uncontrolled seizures and excessive epileptiform abnormalities could worsen the clinical state. Reducing the seizure burden might lead to improved QOL. In DEE, the predictive factors for VNS efficacy remain unclear. Age at seizure onset, age at implantation, duration of epilepsy, and MRI were not predictors of responders post-VNS implantation. It should be emphasized that patients with ES are not suitable candidates for epilepsy surgery. Further investigations are needed to validate our results. Although it has been recently reported that CBD-based AED are an effective alternative to reduce seizures in drug resistant childhood epilepsy, such AEDs are not available in China (as Epidiolex). That is why we have to move one step forward to implement VNS.

## Data availability statement

The raw data supporting the conclusions of this article will be made available by the authors, without undue reservation.

## Ethics statement

The studies involving human participants were reviewed and approved by the Human Ethics Committee of Jinan Children's Hospital. Written informed consent to participate in this study was provided by the participants' legal guardian/next of kin.

## Author contributions

GG and YM analyzed and interpreted the patient data. JS was a major contributor in writing the manuscript. All authors contributed to the article and approved the submitted.

## References

[1] Scheffer IE Berkovic S Capovilla G Connolly MB French J Guilhoto L. ILAE classification of the epilepsies: position paper of the ILAE Commission for Classification and Terminology. Epilepsia. (2017) 58:512–21. 10.1111/epi.13709 28276062 PMC5386840

[2] Morrison-Levy N Borlot F Jain P Whitney R. Early-onset developmental and epileptic encephalopathies of infancy: an overview of the genetic basis and clinical features. Pediatr Neurol. (2021) 116:85–94. 10.1016/j.pediatrneurol.2020.12.001 33515866

[3] Howell KB Freeman JL Mackay MT Fahey MC Archer J Berkovic SF. The severe epilepsy syndromes of infancy: a population-based study. Epilepsia. (2021) 62:358–70. 10.1111/epi.16810 33475165

[4] Yang L Kong Y Dong X Hu L Lin Y Chen X. Clinical and genetic spectrum of a large cohort of children with epilepsy in China. Genet Med. (2019) 21:564–71. 10.1038/s41436-018-0091-8 29930392 PMC6681813

[5] Guerrini R Conti V Mantegazza M Balestrini S Galanopoulou AS Benfenati F. Developmental and epileptic encephalopathies: from genetic heterogeneity to phenotypic continuum. Physiol Rev. (2023) 103:433–513. 10.1152/physrev.00063.2021 35951482 PMC9576177

[6] Ding J Li X Tian H Wang L Guo B Wang Y. Mutation-beyond Dravet syndrome: a systematic review and narrative synthesis. Front Neurol. (2021) 12:743726. 10.3389/fneur.2021.743726 35002916 PMC8739186

[7] Stamberger H Crosiers D Balagura G Bonardi CM Basu A Cantalupo G. Natural history study of STXBP1-developmental and epileptic encephalopathy into adulthood. Neurology. (2022) 99:e221–33. 10.1212/WNL.0000000000200715 35851549 PMC9302932

[8] Specchio N Di Micco V Trivisano M Ferretti A Curatolo P. The epilepsy-autism spectrum disorder phenotype in the era of molecular genetics and precision therapy. Epilepsia. (2022) 63:6–21. 10.1111/epi.17115 34741464

[9] Specchio N Curatolo P. Developmental and epileptic encephalopathies: what we do and do not know. Brain. (2021) 144:32–43. 10.1093/brain/awaa371 33279965

[10] Pressler RM Lagae L. Why we urgently need improved seizure and epilepsy therapies for children and neonates. Neuropharmacology. (2020) 170:107854. 10.1016/j.neuropharm.2019.107854 31751548

[11] Raga S Specchio N Rheims S Wilmshurst JM. Developmental and epileptic encephalopathies: recognition and approaches to care. Epileptic Disord. (2021) 23:40–52. 10.1684/epd.2021.1244 33632673

[12] Dibué M Greco T Spoor JKH Tahir Z Specchio N Hänggi D. Vagus nerve stimulation in patients with Lennox-Gastaut syndrome: a meta-analysis. Acta Neurol Scand. (2021) 143:497–508. 10.1111/ane.13375 33188523 PMC8049065

[13] Ding J Wang L Wang C Gao C Wang F Sun T. Is vagal-nerve stimulation safe during pregnancy? A mini review. Epilepsy Res. (2021) 174:106671. 10.1016/j.eplepsyres.2021.106671 34022523

[14] Mao H Chen Y Ge Q Ye L Cheng H. Short- and long-term response of vagus nerve stimulation therapy in drug-resistant epilepsy: a systematic review and meta-analysis. Neuromodulation. (2022) 25:327–42. 10.1111/ner.13509 35396068

[15] Pérez-Carbonell L Faulkner H Higgins S Koutroumanidis M Leschziner G. Vagus nerve stimulation for drug-resistant epilepsy. Pract Neurol. (2020) 20:189–98. 10.1136/practneurol-2019-002210 31892545

[16] Englot DJ Chang EF Auguste KI. Vagus nerve stimulation for epilepsy: a meta-analysis of efficacy and predictors of response. J Neurosurg. (2011) 115:1248–55. 10.3171/2011.7.JNS11977 21838505

[17] Wang Y Wang D Li D Qian R Fu X Niu C. Improvement of intellectual outcomes in 20 children with refractory epilepsy after individualized surgery. Surg Neurol Int. (2018) 9:203. 10.4103/sni.sni_381_17 30386673 PMC6194735

[18] Huf RL Mamelak A Kneedy-Cayem K. Vagus nerve stimulation therapy: 2-year prospective open-label study of 40 subjects with refractory epilepsy and low IQ who are living in long-term care facilities. Epilepsy Behav. (2005) 6:417–23. 10.1016/j.yebeh.2005.01.009 15820352

[19] Xie H Ma J Ji T Liu Q Cai L Wu Y. Vagus nerve stimulation in children with drug-resistant epilepsy of monogenic etiology. Front Neurol. (2022) 13:951850. 10.3389/fneur.2022.951850 36119689 PMC9475310

[20] Hajtovic S LoPresti MA Zhang L Katlowitz KA Kizek DJ Lam S. The role of vagus nerve stimulation in genetic etiologies of drug-resistant epilepsy: a meta-analysis. J Neurosurg Pediatr. (2022) 18:667–80. 10.3171/2022.1.PEDS222 35303699

[21] Lim Z Wong K Downs J Bebbington K Demarest S Leonard H. Vagus nerve stimulation for the treatment of refractory epilepsy in the CDKL5 Deficiency Disorder. Epilepsy Res. (2018) 146:36–40. 10.1016/j.eplepsyres.2018.07.013 30071384

[22] Zuberi SM Wirrell E Yozawitz E Wilmshurst JM Specchio N Riney K. ILAE classification and definition of epilepsy syndromes with onset in neonates and infants: position statement by the ILAE Task Force on Nosology and Definitions. Epilepsia. (2022) 63:1349–97. 10.1111/epi.17239 35503712

[23] Wirrell EC Nabbout R Scheffer IE Alsaadi T Bogacz A French JA. Methodology for classification and definition of epilepsy syndromes with list of syndromes: report of the ILAE Task Force on Nosology and Definitions. Epilepsia. (2022) 63:1333–48. 10.1111/epi.17237 35503715

[24] Happ HC Carvill GL. A 2020 view on the genetics of developmental and epileptic encephalopathies. Epilepsy Curr. (2020) 20:90–6. 10.1177/1535759720906118 32166973 PMC7160871

[25] Myers KA Johnstone DL Dyment DA. Epilepsy genetics: current knowledge, applications, and future directions. Clin Genet. (2019) 95:95–111. 10.1111/cge.13414 29992546

[26] Hu C Liu D Luo T Wang Y Liu Z. Phenotypic spectrum and long-term outcome of children with genetic early-infantile-onset developmental and epileptic encephalopathy. Epileptic Disord. (2022) 24:343–52. 10.1684/epd.2021.1394 34859793

[27] Tong X Wang X Qin L Zhou J Guan Y Teng P. Vagus nerve stimulation for drug-resistant epilepsy induced by tuberous sclerosis complex. Epilepsy Behav. (2022) 126:108431. 10.1016/j.yebeh.2021.108431 34883463

[28] Ruffoli R Giorgi FS Pizzanelli C Murri L Paparelli A Fornai F. The chemical neuroanatomy of vagus nerve stimulation. J Chem Neuroanat. (2011) 42:288–96. 10.1016/j.jchemneu.2010.12.002 21167932

[29] Wang HJ Tan G Zhu LN Chen D Xu D Chu SS. Predictors of seizure reduction outcome after vagus nerve stimulation in drug-resistant epilepsy. Seizure. (2019) 66:53–60. 10.1016/j.seizure.2019.02.010 30802843

[30] Chrastina J Novák Z Zeman T Kočvarová J Pail M DoleŽalová I. Single-center long-term results of vagus nerve stimulation for epilepsy: a 10-17 year follow-up study. Seizure. (2018) 59:41–7. 10.1016/j.seizure.2018.04.022 29738985

[31] Guo M Wang J Tang C Deng J Zhang J Xiong Z. Vagus nerve stimulation for refractory posttraumatic epilepsy: efficacy and predictors of seizure outcome. Front Neurol. (2022) 13:954509. 10.3389/fneur.2022.954509 35968289 PMC9366668

[32] Liu S Xiong Z Wang J Tang C Deng J Zhang J. Efficacy and potential predictors of vagus nerve stimulation therapy in refractory postencephalitic epilepsy. Ther Adv Chronic Dis. (2022) 13:20406223211066738. 10.1177/20406223211066738 35070253 PMC8771757

[33] Suller Marti A Mirsattari SM MacDougall K Steven DA Parrent A de Ribaupierre S. Vagus nerve stimulation in patients with therapy-resistant generalized epilepsy. Epilepsy Behav. (2020) 111:107253. 10.1016/j.yebeh.2020.107253 32615417

[34] Tsai JD Yang RC Chang MY Fan HC Hung KL Tcns VNS. Vagus nerve stimulation for patients with refractory epilepsy: demographic features and neuropsychological outcomes of the VNS Taiwan child neurology society database. Epilepsy Behav. (2020) 111:107186. 10.1016/j.yebeh.2020.107186 32534423

[35] Knorr C Greuter L Constantini S Fried I Kremer U Datta AN. Subgroup analysis of seizure and cognitive outcome after vagal nerve stimulator implantation in children. Childs Nerv Syst. (2021) 37:243–52. 10.1007/s00381-020-04628-0 32361930

[36] Arya R Greiner HM Lewis A Horn PS Mangano FT Gonsalves C. Predictors of response to vagus nerve stimulation in childhood-onset medically refractory epilepsy. J Child Neurol. (2014) 29:1652–9. 10.1177/0883073813510970 24309242

[37] Yalnizoglu D Ardicli D Bilginer B Konuskan B Karli Oguz K Akalan N. Long-term effects of vagus nerve stimulation in refractory pediatric epilepsy: a single-center experience. Epilepsy Behav. (2020) 110:107147. 10.1016/j.yebeh.2020.107147 32604021

[38] Chrastina J Novak Z Zeman T Dolezalova I Zatloukalova E Brazdil M. Vagus nerve stimulation outcome prediction: from simple parameters to advanced models. Bratisl Lek Listy. (2022) 123:641–7. 10.4149/BLL_2022_103 36039882

[39] Okanishi T Fujimoto A Nishimura M Kanai S Motoi H Homma Y. Insufficient efficacy of vagus nerve stimulation for epileptic spasms and tonic spasms in children with refractory epilepsy. Epilepsy Res. (2018) 140:66–71. 10.1016/j.eplepsyres.2017.12.010 29287185

[40] Japaridze N Muthuraman M Reinicke C Moeller F Anwar AR Mideksa KG. Neuronal networks during burst suppression as revealed by source analysis. PLoS ONE. (2015) 10:e0123807. 10.1371/journal.pone.0123807 25927439 PMC4415810

[41] Lim MJR Fong KY Zheng Y Chua CYK Miny S Lin JB. Vagus nerve stimulation for treatment of drug-resistant epilepsy: a systematic review and meta-analysis. Neurosurg Rev. (2022) 45:2361–73. 10.1007/s10143-022-01757-9 35217961

[42] Zhu S Zhang X Zhou M Kendrick KM Zhao W. Therapeutic applications of transcutaneous auricular vagus nerve stimulation with potential for application in neurodevelopmental or other pediatric disorders. Front Endocrinol. (2022) 13:1000758. 10.3389/fendo.2022.1000758 36313768 PMC9596914

[43] Aniwattanapong D List JJ Ramakrishnan N Bhatti GS Jorge R. Effect of vagus nerve stimulation on attention and working memory in neuropsychiatric disorders: a systematic review. Neuromodulation. (2022) 25:343–55. 10.1016/j.neurom.2021.11.009 35088719

